# Healthcare service utilisation of elderly Ukrainian refugees in Israel: A retrospective cohort study

**DOI:** 10.1080/13814788.2025.2561679

**Published:** 2025-10-15

**Authors:** Limor Adler, Eugene Merzon, Bar Cohen, Michal Shani, Galia Zacay, Pavlo Kolesnyk, Shlomo Vinker

**Affiliations:** ^a^ Maccabi HealthCare Services, Tel Aviv, Israel; ^b^ Department of Family Medicine, Gray’s Faculty of Medical & Health Science, Tel Aviv University, Tel Aviv, Israel; ^c^ Leumit Healthcare Services, Tel Aviv, Israel; ^d^ Adelson School of Medicine, Ariel University, Ariel, Israel; ^e^ Department of Family Medicine Central District, Clalit Health Services, Rehovot, Israel; ^f^ Department of Family Medicine, Meuhedet Health Maintenance Organization, Tel Aviv, Israel; ^g^ LTD “InterFamily,” Uzhgorod, Ukraine; ^h^ Department of Family Medicine and Outpatient Care, Medical Faculty 2, Uzhhorod National University, Uzhgorod, Ukraine

**Keywords:** Refugees, healthcare services, refugee health

## Abstract

**Background:**

The war in Ukraine led to a flood of refugees consisting mainly of women, children and elderly.

**Objectives:**

This study aimed to explore healthcare use by elderly Ukrainian refugees.

**Methods:**

In this retrospective cohort study, we examined patterns of healthcare services used by elderly Ukrainian refugees in Israel between 30 July 2022 and 1 May 2023 (*N* = 2269). We compared them to controls, matched for age and gender, among the general Israeli population (*N* = 2271). We performed Poisson regressions for statistical analysis.

**Results:**

The Ukrainian refugee cohort was predominantly female (77.4%) with a mean age of 71.4 ± 7.1 years. Compared to their controls, the refugees were much less likely to participate in face-to-face, digital and video doctor visits (IRR = 0.838, 0.457 and 0.329, respectively; *p* value < 0.001). Across almost all medical fields (except cardiology), refugees were less likely to have consultations with specialists. Additionally, refugees had fewer emergency room visits (IRR = 0.42, *p* value < 0.001), fewer hospitalisations (IRR = 0.54, *p* value < 0.001) and shorter hospitalisations (IRR = 0.489, *p* value < 0.001).

**Conclusions:**

In a healthcare system with full coverage, Ukrainian refugees were less likely to utilise healthcare services. These findings suggest that refugees may face significant barriers to access and may be underutilising needed care. Healthcare systems should adopt proactive and culturally responsive approaches to address these disparities and ensure equitable access. This study highlights the need for targeted interventions and further research to better understand and reduce healthcare barriers among refugee populations.

## KEY MESSAGES

Elderly Ukrainian refugees used significantly fewer healthcare services than matched Israeli controls, despite having full healthcare coverage.

Refugees had lower rates of primary care consultations, specialist visits, emergency room use, hospitalisations, and shorter hospital stays.

Policymakers should address access barriers – including language and cultural differences – through equity-oriented, refugee-specific healthcare programs.

## Introduction

On 24 February 2022, the Russian invasion of Ukraine triggered the displacement of millions, creating one of the largest refugee crises in recent history. As of December 2023, over six million Ukrainian refugees had been registered globally, mostly in Europe [1]. Israel accepted approximately 14,250 Ukrainian refugees by March 2023, implementing more flexible work and health insurance policies to support them [2].

Providing healthcare for refugees presents complex challenges. Refugees might arrive with medical conditions uncommon in host countries, face health risks during transit and struggle to adapt to unfamiliar healthcare systems. Barriers such as administrative complexity, cultural differences and limited language proficiency often restrict their ability to access care [3]. Refugees struggle to adjust to their host countries’ bureaucracy, administrative processes and cultural differences, which affect their ability to use healthcare services [4–14]. Studies have found that refugees use emergency services more often and outpatient or specialist care less frequently than the general population, despite having more health complaints [7,15,16]. These gaps are sometimes addressed by NGOs, official programs and community networks that help refugees navigate healthcare services [17–19].

Elderly refugees are particularly vulnerable due to multiple chronic diseases, mental health issues and the lasting effects of displacement [20]. They commonly present with multiple chronic diseases and are at higher risk for infectious diseases such as tuberculosis and HIV/AIDS, reflecting their country of origin’s epidemiological profile. Beyond physical ailments, significant mental health challenges are paramount, including elevated rates of depression, anxiety and post-traumatic stress disorder, mainly stemming from exposure to violence, persecution, loss and the inherent stressors of forced migration [21,22]. They frequently experience social isolation, sensory impairments and disabilities, often without formal diagnoses or documentation [23]. Access to care is severely compromised by language and cultural differences, low health literacy regarding host country health systems, financial constraints and fear of discrimination, making communication and navigation of services particularly challenging [21,24].

Few studies about the health needs and proper medical care of Ukrainian refugees since the beginning of the war have been published. Studies from Poland found that Ukrainian refugees commonly presented with infectious and chronic diseases, while facing healthcare access barriers such as language, missing records and legal uncertainties, highlighting the need for system reorganisation [25,26]. The current study aimed to explore healthcare use by elderly Ukrainian refugees compared to matched controls from the general Israeli population.

## Methods

### Study design and population

This retrospective cohort study included all Ukrainian war refugees in Israel who were insured by the state and received medical service from Leumit Health Services (LHS), one of the country’s healthcare maintenance organisations (HMOs), starting from July 2022. LHS insured all Ukrainian refugees over the age of 60 in Israel. This insurance covered all the privileges outlined in the National Insurance Law for Israeli citizens (a detailed explanation on the healthcare system is provided in Box 1). We included only refugees who had been insured for nine months or more when the data was collected. The control group consisted of Israeli LHS members aged 60 years or older, matched to the refugee group by age and gender. To account for shared cultural and linguistic backgrounds, we further limited the control group to individuals born in Ukraine, Russia, or other former Soviet Union countries. Controls were randomly selected from this eligible pool at a 1:1 ratio, ensuring no prior refugee status and continuous membership in the HMO during the study period. LHS has implemented an electronic medical record (EMR) system comprising a comprehensive population and resource use information database. It includes, for example, demographic data, clinical visit records, hospitalisations and emergency department visits. The laboratory tests were performed at a single central laboratory and diagnosis codes were recorded using the International Classification of Diseases, 9th Revision, Clinical Modification. Data collection was performed using IBM Cognos 10.1.1 BI Report Studio software. Query results were downloaded to Microsoft Excel (version 14) spreadsheets for analysis.

Box 1. The healthcare system in Israel.

The Israeli healthcare system is based on healthcare coverage provided by a specific healthcare maintenance organisation, which offers primary care and specialist consultations in the community and which directly pays for other services provided at public or affiliated hospitals. Therefore, it serves as the primary ‘case manager’ for each patient’s medical needs, unless these needs are addressed privately and not subsequently documented. Upon arrival in Israel, refugees from Ukraine who are over 60 years of age were automatically provided coverage by a specific healthcare maintenance organisation (LHS Healthcare Services). Their coverage allowed them access to primary care resources, consults with specialists, emergency room visits and hospitalisations as needed.

### Variables

The following variables were collected for both groups during the nine-month study period: number of visits to a primary care physician (PCPs) and visit domain, i.e. phone, in-person, or digital visit; number of visits to an emergency department; number of visits to specialists (urology, gynaecology, endocrinology, gastroenterology, general surgery, orthopaedic surgery, dermatology, ophthalmology neurology and psychiatry); number of hospitalisation; referrals for elective surgery; and referrals for dialysis. Additionally, we collected basic demographic data, including age and gender (used for matching).

### Statistical analysis

Descriptive statistics were used to summarise the characteristics of the study population. Continuous variables were presented as means and standard deviations (SD), while categorical variables were presented as counts and percentages.

### Comparative analysis

Statistical analyses were performed using R software version 4.0.2 (R Foundation for Statistical Computing, Vienna, Austria) and Intercooled Stata version 9.1 (StataCorp, College Station, TX, USA). Sociodemographic differences between Ukrainian refugees and matched controls were assessed using Student’s t-tests for continuous variables and Fisher’s exact χ^2^ tests for categorical variables. Two-sided tests with a significance level of 0.05 were applied.

### Regression modelling

To assess differences in healthcare utilisation (count outcomes), we used Poisson regression models, reporting incidence rate ratios (IRRs) with 95% confidence intervals (CIs). An offset term was included in each model to account for the follow-up time, ensuring rate-based comparisons. All models were adjusted for age and sex.

### Handling of missing data and multiple comparisons

Missing data were assessed for each variable. For variables with <10% missingness, complete-case analysis was used. For variables with more than 10% missingness, multiple imputation via chained equations (MICE) was applied under the assumption that the data were missing at random (MAR). To account for multiple comparisons across outcome domains, we applied the Benjamini-Hochberg false discovery rate (FDR) correction method to reduce the risk of Type I error.

## Results

### Participants

This study compared 2269 refugees with 2271 matched non-refugee Israeli controls. The mean age in the refugee group was 71.4 (± 7.09), and reflecting the characteristics of the general Ukrainian refugee population, most of the patients were women (77.4%).

### Univariate analysis

#### Utilisation of primary care services

Compared to their matched controls, refugees were less likely to use digital and video visits with their PCPs (IRR = 0.46 and 0.33, respectively, *p* value < 0.001) and somewhat less likely to use phone or in-person visits (IRR = 0.838 and 0.888, respectively, *p* value < 0.001). The duration of all visits with PCPs during the study period was shorter for refugees (35.4 vs. 37.5 min, *p* value < 0.001).

#### Specialist care utilisation

Refugees were less likely to have consultations with specialists, including psychiatrists (IRR = 0.315, *p* value < 0.001), dermatologists (IRR = 0.352, *p* value < 0.001), endocrinologists (IRR = 0.485, *p* value < 0.001), orthopaedics (IRR = 0.542, *p* value < 0.001), ophthalmologists (IRR = 0.592, *p* value < 0.001), gastroenterologists (IRR = 0.529, *p* value < 0.001), general surgeons (IRR = 0.607, *p* value < 0.001) and neurologists (IRR = 0.716, *p* value < 0.001) (Table 1). Consultations with OB/GYN were not significantly different between the two groups (IRR = 0.800, *p* value = 0.740). The exception was cardiologists, whom refugees consulted more frequently than the control population (IRR = 1.21, *p* value = 0.011).

**Table 1.** Descriptive statistics of study population and controls.

	Cases *n* = 2269	Matched controls *n* = 2271	IRR (95%CI)	*p* value
Age, mean ± *SD*	71.4 ± 7.09	71.4 ± 7.76	1.00 [0.99–1.00]	0.951
Women, *N* (%)	1757 (77.37%)	1756 (77.39%)	1.00 [0.93–1.07]	0.993
Digital visits (mean ± *SD*)	0.66 ± 1.93	1.45 ± 2.44	0.46 [0.43–0.49]	<0.001
Video visits (mean ± *SD*)	0.01 ± 0.12	0.03 ± 0.25	0.33 [0.20–0.53]	<0.001
Phone visits (mean ± *SD*)	1.03 ± 2.38	1.16 ± 2.16	0.89 [0.84–0.94]	<0.001
Primary care physician visits (mean ± *SD*)	5.49 ± 5.55	5.97 ± 5.43	0.84 [0.82–0.86]	<0.001
Primary care physician visits (minutes ± *SD*)	35.41 ± 47.11	37.53 ± 43.08	0.94 [0.93–0.95]	<0.001
Ob/Gyn visits (mean ± *SD*)	0.001 ± 0.05	0.002 ± 0.07	0.80 [0.21–2.98]	0.740
Endocrinologist visits (mean ± *SD*)	0.094 ± 0.49	0.194 ± 0.65	0.48 [0.41–0.57]	<0.001
Gastroenterologist visits (mean ± *SD*)	0.12 ± 0.58	0.22 ± 0.72	0.53 [0.46–0.61]	<0.001
Orthopaedic surgeon visits (mean ± *SD*)	0.30 ± 0.92	0.55 ± 1.19	0.54 [0.49–0.60]	<0.001
General surgeon visits (mean ± *SD*)	0.10 ± 0.49	0.170 ± 0.56	0.61 [0.52–0.71]	<0.001
Dermatologist visits (mean ± *SD*)	0.11 ± 0.46	0.31 ± 0.76	0.35 [0.30–0.41]	<0.001
Ophthalmologist visits (mean ± *SD*)	0.29 ± 0.75	0.50 ± 0.96	0.59 [0.54–0.65]	<0.001
Neurologist visits (mean ± *SD*)	0.09 ± 0.40	0.13 ± 0.48	0.72 [0.60–0.85]	<0.001
Psychiatrist visits (mean ± *SD*)	0.02 ± 0.19	0.05 ± 0.37	0.31 [0.22–0.45]	<0.001
Cardiologist visits (mean ± *SD*)	0.17 ± 0.63	0.14 ± 0.50	1.21 [1.04–1.40]	0.011
Emergency department visits (mean ± *SD*)	0.06 + 0.30	0.14 ± 0.47	0.42 [0.35–0.52]	<0.001
Hospitalisation (mean ± *SD*)	0.13 + 0.70	0.24 + 1.08	0.54 [0.47–0.63]	<0.001
Hospital stay days (mean ± *SD*)	0.04 + 0.24	0.07 + 0.83	0.49 [0.37–0.64]	<0.001
Dialysis Tx (mean ± *SD*)	0.18 + 5.41	0.31 + 7.37	0.16 [0.13–0.20]	<0.001
Surgeries (mean ± *SD*)	0.07 + 0.32	0.11 + 0.44	0.61 [0.50–0.75]	<0.001

Note: IRR: Incidence rate ratio; *SD*: Standard deviation; Tx: Treatments.

#### Utilisation of emergency and hospital services among refugees

**Figure 1.**
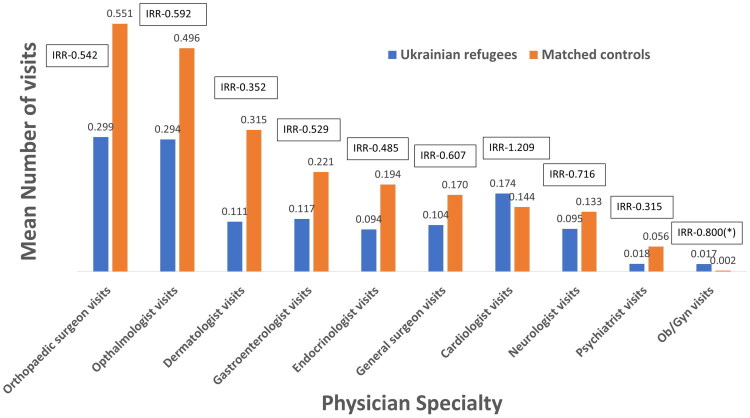
Mean number of visits to specialists per patient. *Notes:* This figure shows the mean number of specialist visits among Ukrainian refugees and their matched controls during the study period. The boxes above each column display the incidence rate ratio (IRR) for the comparison. An asterisk (*) indicates non-significant results, while all other comparisons are statistically significant (*p* < 0.05).

**Figure 2.**
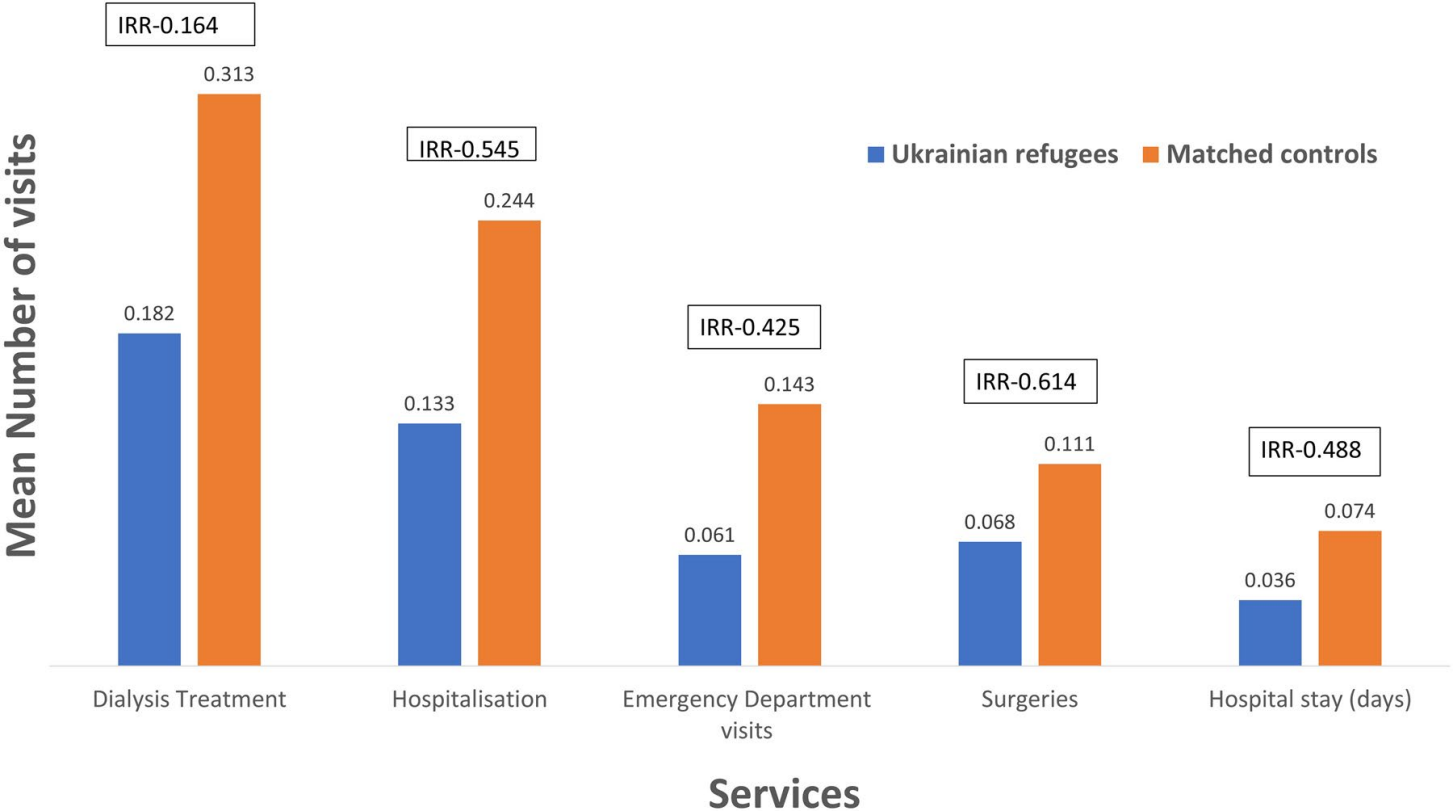
Healthcare services use: mean number of visits per patient. This figure displays the mean number of emergency department visits, hospitalisations, hospital stay days, dialysis treatments and surgeries (of any type) among Ukrainian refugees and their matched controls during the study period. The boxes above each column display the incidence rate ratio (IRR) for the comparison. All results are statistically significant (*p* < 0.05).

Refugees had fewer emergency room visits (IRR = 0.42, *p* value < 0.001), fewer hospitalisations (IRR = 0.54, *p* value < 0.001), shorter hospitalisations on average (IRR = 0.49, *p* value < 0.001) and fewer referrals to dialysis (IRR = 0.16, *p* value < 0.001) and elective surgeries (IRR = 0.61, *p* value < 0.001) (Table 1, Figure 1 and Figure 2).

## Discussion

### Main findings

This study found that Ukrainian refugees in Israel used healthcare services less than matched controls, despite having access to universal health coverage. This included primary care visits, specialist consultations, emergency room visits, hospitalisations and use of remote services such as digital, phone and video consultations.

### Interpretation

Refugees frequently demonstrate lower utilisation of healthcare services despite often having increased health needs stemming from pre-migration trauma and post-migration stressors [16]. Our study found consistent underutilisation of services across most domains.

This underutilisation is a complex issue driven by a combination of systemic, cultural and individual barriers. A prominent barrier is the linguistic and communication challenges, characterised by a significant lack of professional interpreters and adequate language services [7,16,24,27–29]. This directly impairs effective patient-provider communication, hindering refugees’ ability to express symptoms, understand medical information and comprehend treatment plans. Over-reliance on unqualified or family interpreters can also lead to inaccuracies and discomfort.

Many refugees are unfamiliar with navigating appointment systems, referral pathways and insurance processes, particularly in a new language [7,16,24,28]. Healthcare providers themselves may also lack sufficient information on patients’ health histories due to an absence of shared medical records across systems or countries.

Beyond communication and information, cultural differences and health-seeking behaviours pose significant impediments, encompassing varying perceptions of illness and a general stigma associated with seeking formal care [7,16,27]. Administrative and systemic barriers are also critical, as legal status often determines the scope of accessible health and social services. Complex and lengthy procedures for obtaining entitlements create significant hurdles [7,16,24,27,29]. Even in a system with universal coverage, indirect costs—such as transport, lost wages, or uncovered medications—can present real obstacles.

These multifaceted barriers, coupled with social isolation, challenging living conditions and the burden of pre-existing or new health concerns from their journey, collectively contribute to refugees observed lower overall healthcare utilisation. This leads to a persistent gap between their diverse health needs and the healthcare they receive.

Underutilisation was observed across both primary care and specialist consultations, except for cardiologist visits. The exception observed in cardiology is notable and may reflect the health profile of older refugees, many of whom may have pre-existing cardiovascular conditions requiring ongoing monitoring, medication adjustments, or diagnostic testing. Additionally, Israel’s healthcare system includes a relatively high number of Russian-speaking clinicians, which may facilitate communication and increase comfort in some cases.

The lower use of emergency and hospitalisations in our study contrasts with findings in other refugee populations and may reflect delayed care-seeking, cultural differences, or systemic factors that warrant further study [7,15].

The finding that refugees were less likely to use non-traditional doctor visits (digital, phone and video) may be attributed to limited digital literacy, unfamiliarity with remote care models and cultural preferences for in-person interactions. Language barriers also play a role, as having a family member assist with interpretation is often more feasible during in-person visits. A literature review found limited information on immigrants’ and refugees’ experiences with virtual care [30]. Given the relatively old age of the refugee population, many may lack the skills or confidence to use digital tools and support from younger relatives may be unavailable. Future efforts should focus on adapting telemedicine tools to meet the cultural and technological needs of older and displaced populations.

### Strengths and limitations

The main strength of this study lies in its comprehensive and reliable dataset from a centralised healthcare system, capturing all medical consultations, treatments and services covered by Israel’s HMO. This allowed a detailed analysis of healthcare use patterns among elderly Ukrainian refugees. The use of matched controls from the general Israeli population, as well as an additional analysis using controls born in Russia, Ukraine, or the former Soviet Union, further strengthens the study by helping to isolate refugee-specific effects.

However, several limitations should be noted. As a retrospective observational study, it is subject to potential bias and unmeasured confounding. While matching helps reduce bias, we did not account for key variables such as comorbidities, education, socioeconomic status, health literacy, or psychosocial stressors that may affect healthcare utilisation. Relying on administrative data also limits insight into clinical context or undocumented conditions. The 9-month follow-up period may not capture longer-term patterns, especially given the time required to receive referrals or adjust to a new healthcare system. Nonetheless, this period offers valuable insight into initial healthcare-seeking behaviour upon arrival, which may evolve over time with greater assimilation.

### Implications

Improving healthcare access and utilisation for refugees requires targeted interventions and supportive policies. Understanding their unique needs is essential for delivering appropriate care. A key first step is proactive outreach, including clear communication in refugees’ native languages. This can be facilitated through professional translation services or bilingual staff.

Educating refugees on how to navigate the healthcare system empowers them to seek care effectively. To address structural barriers, health systems should provide culturally adapted services, streamline administrative processes and expand digital health options – especially for those with mobility or transportation limitations.

For elderly refugees, interventions should address age-related challenges such as sensory impairments, chronic disease monitoring and functional limitations. Geriatric care, assistance with appointment scheduling and transportation support may reduce underutilisation in this group.

The findings of this study can inform future research on optimising refugee care and guide inclusive clinical practices. At the policy level, these results highlight the need for healthcare models that prioritise chronic disease management, mental health support and preventive care. Mandating cultural competence training and allocating dedicated resources to refugee health can enhance system responsiveness. By addressing these gaps, healthcare systems can reduce disparities and promote equity for refugee populations.

## Conclusions

Despite full healthcare coverage, elderly Ukrainian refugees in Israel used significantly fewer healthcare services than matched controls. This underutilisation spanned primary care, specialist consultations, emergency services, hospitalisations, dialysis and elective surgeries. These findings suggest that formal access alone is insufficient; refugees face persistent barriers that may compromise care. Healthcare providers and policymakers must adopt proactive, culturally informed strategies to reduce these gaps. Priorities include health system orientation for refugees, language-appropriate communication and matching patients with providers sensitive to their needs. Future research should focus on the early identification of refugee health needs and interventions that improve access to and utilisation of services across various care settings.
